# Evaluation of the effects of whitening toothpaste containing nanohydroxyapatite on surface roughness and color change in restorative materials

**DOI:** 10.7717/peerj.15692

**Published:** 2023-07-14

**Authors:** Burak Dayı, Fikri Öcal

**Affiliations:** Department of Restorative Dentistry, Faculty of Dentistry, Inonu University, Malatya, Turkey

**Keywords:** Bleaching, Coloration, Dental composite, Profilometer, Restoration bleaching

## Abstract

**Background:**

The effects of whitening toothpastes containing nanohydroxyapatite on the surfaces of restorative materials are not well known. This study evaluated the changes in surface roughness and color of coffee-stained restorative materials after brushing with nanohydroxyapatite and other whitening toothpastes.

**Methods:**

Disc-shaped specimens were formed using microhybrid, nanohybrid, and supra-nano-filled composite (*n* = 30) and stained with a coffee solution. A brushing simulation was applied with toothpastes containing nanohydroxyapatite (Dentiste Plus White), perlite (Signal White System) and hydrogen peroxide (Colgate Optic White) for 7, 15, and 30 days. Color and surface roughness measurements were taken before and after brushing. Color change (∆E_00_) was calculated using the CIEDE2000 system.

**Results:**

Supra-nano-filled composite-Dentiste Plus White, supra-nano-filled composite-Colgate Optic White, and nanohybrid composite-Signal White System were the groups with the greatest color change observed on the 7th, 15th, and 30th days, respectively. The smallest color change was seen in the microhybrid composite-Signal White System, microhybrid composite-Dentiste Plus White, and nanohybrid composite-Dentiste Plus White groups on the 7th, 15th, and 30th days, respectively. No significant surface roughness changes were observed in the nanohybrid composite-Dentiste Plus White, supra-nano-filled composite-Colgate Optic White, supra-nano-filled composite-Dentiste Plus White, or supra-nano-filled composite-Signal White System groups.

**Conclusions:**

Whitening toothpaste containing nanohydroxyapatite provided a high degree of color change in the short term and did not create significant surface roughness in nanohybrid or supra-nano-filled composites.

## Introduction

Today, patient demand for esthetic restorations has led to an increase in the use of resin composites and studies on these materials. To provide an aesthetic appearance in restorative dentistry, restorations resembling natural teeth should be applied, and this similarity should be maintained for a long time. In this context, the color stability of the composite restoration and the detection of discoloration are important criteria in evaluating material success (Roselino et al., 2013). One of the important factors required for composite restoration to preserve its color for a long time is the maintenance of surface smoothness (Kocaagaoglu et al., 2017). Tooth brushing time, certain toothpaste types, and applied force can negatively affect the long-term durability of the surface shine of composite restorations (de Moraes Rego Roselino et al., 2019).

Since resin composites were first developed, efforts have been made to enhance their clinical efficacy. Whereas resin composite research is mostly focused on the creation of novel monomers, investigations on filler content concentrate on particle size and silanization (Atai et al., 2004; Lu et al., 2005). Nanotechnology examines materials in the range of about 0.1–100 nm and is known as the manufacture and manipulation of these structures using different physical or chemical processes (Beun et al., 2007). Whereas the particle size in hybrid composites is 8–30 µm and around 0.7–3.6 µm in microhybrid composites, restorative materials with particle sizes ranging from 5–100 nm have been recently developed (Sivakumar et al., 2016; Moszner & Klapdohr, 2004). Composites containing supra-nano particles have been created using the sol-gel process, which adjusts the diameter of the fillers while also changing their refractive index (Perez et al., 2016). The manufacturer states that the particles in the filler content are spherical, allowing it to maintain its brightness and exhibit a realistic opalescence (Tokuyama Dental, 2011).

Toothpastes with whitening properties used to clean the teeth and make them look whiter may contain abrasives such as hydrated silica, calcium carbonate, calcium pyro-phosphate, perlite, and sodium bicarbonate (Epple, Meyer & Enax, 2019). In addition to abrasives, toothpastes containing chemicals (*e.g*., phosphates) and dyes (*e.g*., blue covariate) are also available (Sarembe et al., 2020). Research has focused on toothpastes that whiten while causing minimal abrasion to the surface of teeth and restorative materials (Vaz et al., 2019; Schlafer et al., 2021). Whitening toothpastes containing nano-hydroxyapatite (n-HAP), a calcium phosphate derivative, is among these.

Hydroxyapatite (HAP) is morphologically similar to the apatite crystal found in human tooth enamel. In addition to the biocompatible and biomimetic properties of this molecule, its whitening properties have attracted the attention of dentists (Shang, Kaisarly & Kunzelmann, 2022). Many researchers have applied hydroxyapatite to teeth in various forms and stated that it has a whitening effect (Onuma, Yamagishi & Oyane, 2005; Kim et al., 2006). Jin et al. (2013) stated that HAP whitens not only because it is white, but also because a freshly formed thin coating of HAP is thought to help diffuse reflections on the tooth surface, making the teeth look brighter.

Knowing the discoloration and abrasive effects of whitening toothpastes with different active ingredients, which are frequently preferred by patients for teeth whitening, on teeth and composite restorations can help physicians to recommend a suitable whitening toothpaste for the appropriate patient. In this way, patients may minimize the roughness that may occur on the restoration surfaces and the negative effects of this situation, while using an effective whitening toothpaste for removing and preventing discoloration in composite restorations. There are many studies in the literature on the effects of whitening toothpastes on teeth and composite restorations (Roselino et al., 2013; de Moraes Rego Roselino et al., 2019; Vaz et al., 2019; Schlafer et al., 2021; Shang, Kaisarly & Kunzelmann, 2022; Kim et al., 2006; Jin et al., 2013; Yilmaz et al., 2021; Dos Santos et al., 2019; Celik & Iscan Yapar, 2021). However, as far as we know, there has been no study comparing the effectiveness of n-HAP-containing toothpastes in removing the discoloration that may occur on the surfaces of composite restorative materials and their effects on the change in surface roughness with other whitening toothpastes. The null hypothesis tested is that whitening toothpaste including n-HAP will cause similar discoloration changes on the surfaces of different types of resin composites after different brushing times and will cause similar roughness changes on the surfaces of different types of resin composites at the end of the brushing procedure compared to other whitening toothpastes.

## Materials and Methods

This study is an *in vitro* study using three different types of whitening toothpastes and three different types of composites.

### Preparation of samples and power analysis

*A priori* power analysis using WSSPAS web-based software revealed that the minimum sample size necessary to detect a significant difference was 25 in each group (75 in total), considering type I error (alfa) of 0.05, power (1-beta) of 0.9 and effect size of 1.05 (Arslan et al., 2018). The current study enrolled 90 composite materials (30 in each main material group) to increase the power of the current research. Disc-shaped specimens with a thickness of 2 mm and a width of 10 mm were prepared using microhybrid composite (3M Z250 Filtek; 3M ESPE, St. Paul, MN, USA), nanohybrid composite (Polofil, Voco, Cuxhaven, Germany), and supra-nano-filled composite (Estelite Sigma Quick, Tokuyama Dental, Tokyo, Japan) (*n* = 30). All preparation methods were carried out according to the manufacturer’s instructions. The content of the composite materials is shown in Table 1.

**Table 1:** Restorative materials used in the study.

Composite	Type	Compound	Manufacturer
Filtek Z250	Microhybrid	Matrix: Bis GMA, Bis EMA, UDMA, TEGDMA Filler: Zirconia/Silica Filler size: 0.01–3.5 µm	3M ESPE, St. Paul, MN, USA
Polofil NHT	Nanohybrid	Matrix: Bis GMA, UDMA, TEGDMA Filler: Glass ceramic Filler size: 0.01–0.1 µm	Voco, Cuxhaven, Germany
Estelite sigma quick	Supra-nano-filled	Matrix: Bis GMA, UDMA, TEGDMA Filler: Silicon dioxide, Zirconium dioxide, Titanium dioxide Filler size: 0.1–0.3 µm	Tokuyama Dental, Tokyo, Japan

**Note:**

Bis-GMA, 2,2-bis[4-(2-hydroxy-3-methacryloxypropoxy)phenyl]propane; Bis-EMA, bis-phenol A ethoxylated dimethacrylate; UDMA, 1,6-bis(methacrylyloxy-2-ethoxycabonylamino)-2,4,4-trimethylhexane/urethane dimethacrylate; TEGDMA, triethyleneglycol dimethacrylate.

Composite resins were placed in a specially prepared Teflon mold (2 × 10 mm), and after the excess was removed by pressing lightly with transparent tape (Mylar strips), the samples were polymerized for 20 s with an LED light device (Guilin Woodpecker Medical Instrument Co., Guilin, China) emitting light at a wavelength of 470 nm (light intensity: 1,200 mW/cm^2^). Then, extra-coarse, coarse/medium, fine, and extra-fine polishing discs (OptiDisc, Kerr, FL, USA) were used consecutively for 15 s to polish the surfaces of the samples. New discs were used for each sample. All samples were stored in distilled water at 37 °C for 24 h.

The initial surface roughness (Ra_0_) of each sample was measured using a mechanical profilometer (Mitutoyo SJ-210; Mitutoyo, Kawasaki, Japan), and basic color values were measured using a spectrophotometer (VITA Easyshade V, VITA Zahnfabrik, Bad Säckingen, Germany). Next, the samples were kept in a specially prepared coffee solution (Nescafe Gold, Nestle, Vevey, Switzerland) at room temperature for 5 days. The coffee solution was obtained by mixing 3 g of coffee powder and 150 ml of boiling water according to the manufacturer’s instructions, and the solution was renewed daily. In previous studies, it was stated that the average daily coffee consumption in the population was 3.2 cups, and the average coffee consumption time was 15 min (Yu et al., 2018). The exposure time of 5 days, therefore, corresponded to approximately 5 months.

### Brushing procedure

Colored composite samples in three main material groups were divided into three subgroups (*n* = 10): three different whitening toothpastes containing nanohydroxyapatite (Dentiste Plus White, Dentiste, Thailand), perlite (Signal White System, Signal Unilever, London, UK), and hydrogen peroxide (Colgate Optic White, Colgate-Palmolive, New York, NY, USA) were used to brush samples with an electric toothbrush (Oral B Clean DB04; Procter & Gamble, Cincinnati, OH, USA). The compositions of the toothpastes are listed in Table 2. Assuming that there are 28 teeth in the mouth, the maximum contact time of a tooth was determined as 10 s, based on the recommended 2 min of brushing twice a day (Addy et al., 2002). Thus, the groups were subjected to brushing simulation for 70, 150, and 300 s (7, 15, and 30 days, respectively). Each brushing session was performed by the same operator to ensure standardization.

**Table 2:** Whitening toothpastes used in the study.

Toothpaste	Compound	Manufacturer
Dentiste plus white	Sorbitol, Silicon Dioxide, Deionized Water, Xylitol, Glicerin, Green Tea Extract, Sodyum Lauryl Sulfate, PVP, Hydroxiapatite, Sodium Tripolyphosphate, Cellulose Gum, Titanium Dioxide, Sodium Benzoate, Sodium Saccharin, Menthol, Ascorbic Acid, Mica, Mentha Piperita Oil, Eucalyptus Globulus Leaf Oil, Eugenia Caryophyllus Flowe Oil, Salvia Officinalis Extract, Anthemis Nobilis Extract, Foeniculum Vulgare Extract, Glycyrrnhiza Grabra Extract, Cinnamomum Cassia Bark Extract, Cl 42090	Dentiste, Thailand
Colgate optic white	Glycerin, Propylene Glycol, Calcium Pyrophosphate, PEG/PPG-116/66 Copolymer, PVP, PEG-12, Pentasodium Triphosphate, Sodium Lauryl Sulfate, Silica, Aroma, Sodium Monofluorophosphate, Disodium Pyrophosphate, Sodium Saccharin, Hydrogen Peroxide, Limonene, Cl74160	Colgate-Palmolive, USA
Signal white system	Calcium Carbonate, Aqua, Sorbitol, Hydrated Silica, Sodium Lauryl Sulfate, Sodium Monofluorophosphate, Aroma, Cellulose Gum, Trisodium Phosphate, Perlite, Sodium Saccharin, Benzyl Alcohol, Glycerin, Sodium Laureth Sulfate, Cl74160	Signal Unilever, UK

**Note:**

PVP, polyvinylpyrrolidone; PEG, polyethylene glycol; PPG, polypropylene glycol.

### Surface roughness and color measurement

The final surface roughness (Ra_1_) measurements were repeated at three different points of each sample with a mechanical profilometer (Mitutoyo SJ-210; Mitutoyo, Kawasaki, Japan), and average values were recorded. Color measurements of all samples were performed by the same operator using a spectrophotometer (VITA Easyshade V, VITA Zahnfabrik, Bad Säckingen, Germany) on a standard white background with the help of D65 illumination and CIEDE2000 color coordinates. The color coordinates of the samples were measured at the beginning of the brushing simulation (T0), after the coloring process (T1), at the end of the 7-day (T2), 15-day (T3), and 30-day (T4) brushing simulation applications. The samples were cleaned under running water after each brushing cycle, and color measurements were conducted immediately after washing. The CIEDE2000 formula was used to calculate the color changes (Paravina et al., 2015). The perceptibility threshold was determined as ΔE_00_ > 0.8 units and the clinical acceptability threshold as ΔE_00_ ≤ 1.8 units (Paravina et al., 2015; Tinastepe et al., 2021).

${\rm \Delta}{\rm {E}}_{00}={\left( {\displaystyle{{{\rm \Delta} {\rm{L}}^{\prime}} \over {{k_L}{S_L}}}} \right)^2} + {\left( {\displaystyle{{{\rm \Delta}{C}^{\prime}} \over {{k_C}{S_C}}}} \right)^2} + \; {\left( {\displaystyle{{{\rm \Delta}{H}^{\prime}} \over {{k_H}{S_H}}}} \right)^{2\; }} + {R_T}\left( {\displaystyle{{{\rm \Delta}{C}^{\prime}} \over {{k_C}{S_C}}}} \right)\; \left( {\displaystyle{{{\rm \Delta}{H}^{\prime}} \over {{k_H}{S_H}}}} \right)$

In the formula: ΔL’, ΔC’ and ΔH’ denote the variations in lightness, chroma, and hue differences between the two samples. The weighting functions for the lightness, chroma, and hue parameters are S_L_, S_C_ and S_H_. According to previous research, K_L_, K_C_ and K_H_ are the regulated variables with regard to the many observed metrics (Lee, 2005; Luo, Cui & Rigg, 2001).

### Biostatistical analysis

The conformance of the quantitative data to the normal distribution was assessed with the Shapiro-Wilk test. Since the quantitative data did not show normal distribution (*p* > 0.05), the data were summarized with median and interquartile range (IQR). Kruskal-Wallis H test was employed for intergroup comparison of the data, and the Conover test was performed for *post-hoc* analysis. Wilcoxon signed-rank test was utilized to compare two repeated measurements. A two-way permutational multivariate analysis of variance (PERMANOVA) test was employed for color measurements related to the first factor (the groups: microhybrid composite-Dentiste Plus White, microhybrid composite-Colgate Optic White, microhybrid composite-Signal White System, nanohybrid composite-Dentiste Plus White, nanohybrid composite-Colgate Optic White, nanohybrid composite-Signal White System, supra-nano-filled composite-Dentiste Plus White, supra-nano-filled composite-Colgate Optic White, and supra-nano-filled composite-Signal White System) and the second factor (the measurements: T_0_–T_1_ (ΔE_00_), T_1_–T_2_ (ΔE1), T_1_–T_3_ (ΔE2) and T_1_–T_4_ (ΔE3)). The PERMANOVA test was performed on Euclidean similarity matrices, and the residuals were achieved from a reduced model having 9,999 permutations. All *p* < 0.05 values were regarded as significant in all analyses. Multiple linear regression analysis with stepwise variable selection was performed to examine independent predictors (composites and kinds of toothpaste) of color, Ra_0_, and Ra_1_ results. The significance of multiple linear regression models was evaluated by analysis of variance (ANOVA) test (*p* < 0.05), and the related statistics, including a 95% confidence interval of lower and upper bounds for the coefficients, R-squared, *etc*., were also reported. Cohen d effect size was calculated to examine the clinical effects of significant differences for Ra measurements. In addition, box whisker plots were drawn for Ra and color results according to composite and toothpaste types. American Psychological Association (APA) 6.0 style was utilized to report the results of the statistical analyses (Yagin et al., 2021). All data analyses were carried out using PAST 4.10 and IBM SPSS Statistics 28.0 for Windows (New York, NY, USA).

## Results

### Color change results

The color changes of the composite material groups after coloring with coffee for 5 days (ΔE_00_) are shown in Table 3. Among the main material groups, the group with the most coloration after exposure to coffee was the microhybrid composite group (ΔE_00_ = 5.24). Color change in all materials exposed to coffee was clinically unacceptable (ΔE_00_ > 1.8), and this change was not statistically significant (*p* = 0.123).

**Table 3:** Color changes in ΔE_00_ (T_0_–T_1_) values in the main material groups.

Variable*	Material type			*p*-value^#^
	Microhybrid composite	Nanohybrid composite	Supra-nano-filled composite	
ΔE_00_ (T_0–_T_1_ )	5.248 (1.499)	4.395 (2.277)	4.158 (2.245)	0.123

**Notes:**

* The data are summarized as ‘median (interquartile range)’.

# Kruskal Wallis H test.

**Figure 1:**
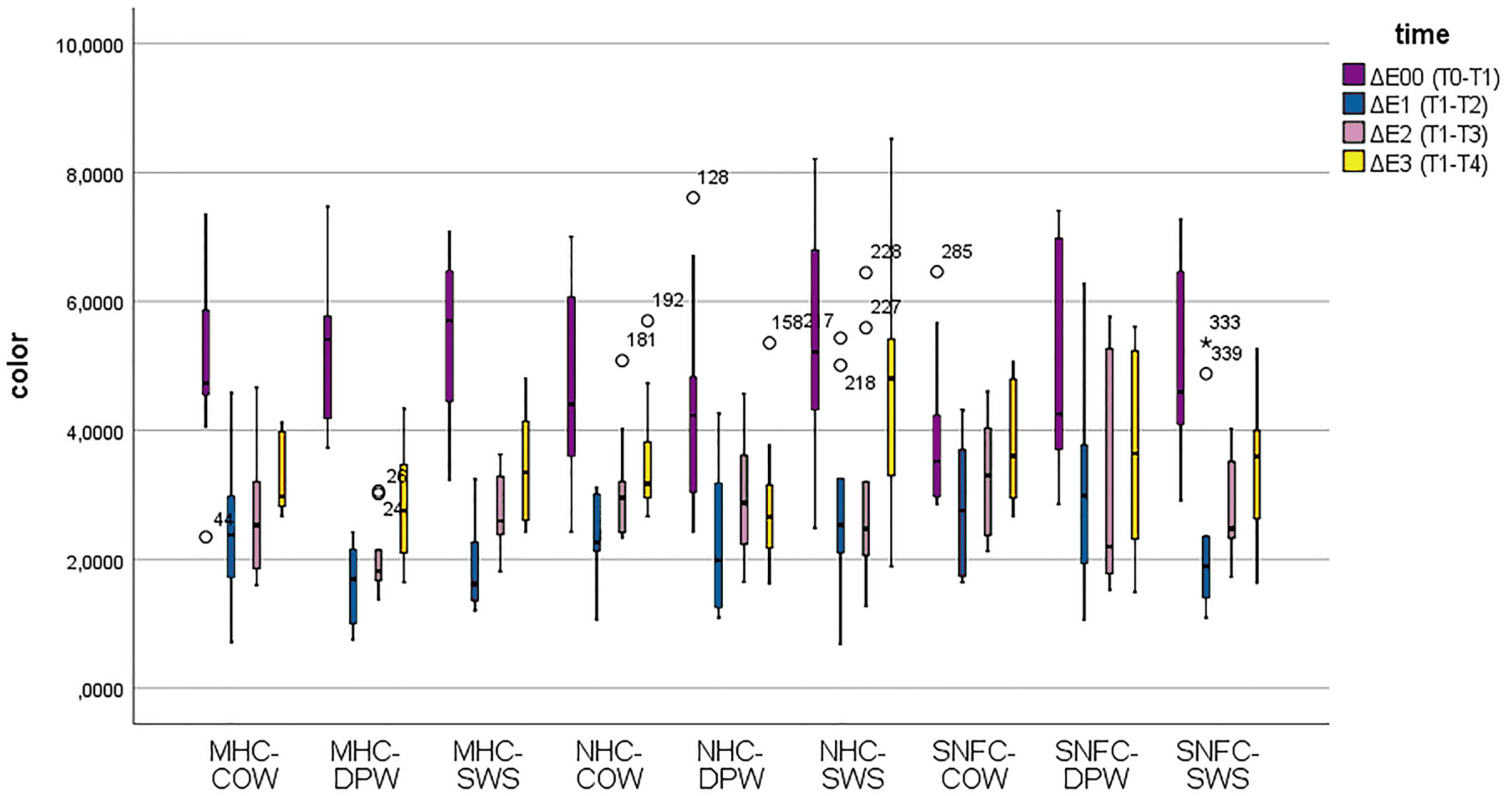
Box-plot graphs for color change data of the MHC-COW, microhybrid composite-colgate optic white; MHC-DPW, microhybrid composite-dentiste plus white; MHC-SWS, microhybrid composite-signal white system; NHC-COW, nanohybrid composite-colgate optic white; NHC-DPW, nanohybrid composite-dentiste plus white; NHC-SWS, nanohybrid composite-signal white system; SNFC-COW, supra-nano-filled composite-colgate optic white; SNFC-DPW, supra-nano-filled composite-dentiste plus white and SNFC-SWS, supra-nano-filled composite-signal white system composite-toothpaste subgroups.

Table 4 shows the PERMANOVA test results of color changes between time periods of the subgroups. The distribution of the variables is given with a box-plot graph (Fig. 1). According to the findings; when the ΔE1 values, which express the color change at the end of the 7th day, were examined, the most color change was seen with the Dentiste Plus White applied to the supra-nano-filled composite group. The least color change was observed after Signal White System applied to the microhybrid composite group. When the ΔE2 values, which express the color change at the end of the 15th day, were examined, the most color change was seen after the Colgate Optic White toothpaste was applied to the supra-nano-filled composite group. The least color change was observed after Dentiste Plus White was applied to microhybrid composite group. When the ΔE3 values, expressing the color change at the end of the 30th day were examined, the group with the most color change was the nanohybrid composite group in which Signal White System was applied. The least color change was seen in the nanohybrid composite group in which Dentiste Plus White was applied.

**Table 4:** PERMANOVA test results for color change measurements.

Groups	Median (IQR)	Between measurements	Between groups	Interaction
		F value	F value	
		*p*_*1*_ value	*p*_*2*_ value	
MHC-DPW ΔE_00_	5.211 (1.4)	F = 70.19; *p*_*1*_ < 0.001 *Post-hoc* analyses (only significant pairwise comparisons) (ΔE_00_ and ΔE1)* (ΔE_00_ and ΔE2)* (ΔE_00_ and ΔE3)* (ΔE1 and ΔE2)* (ΔE1 and ΔE3)* (ΔE2 and ΔE3)*	F = 2.46; *p*_*2*_ = 0.01 *Post-hoc* analyses (only significant pairwise comparisons) (MHC-DPW and NHC-SWS)* (MHC-DPW and SNFC-DPW)* (NHC-DPW and NHC-SWS)*	F = 1.17; *p* = 0.25
MHC-DPW ΔE1	1.693 (1.067)			
MHC-DPW ΔE2	1.814 (0.423)			
MHC-DPW ΔE3	2.751 (1.236)			
MHC-COW ΔE_00_	4.73 (1.118)			
MHC-COW ΔE1	2.374 (1.071)			
MHC-COW ΔE2	2.53 (1.279)			
MHC-COW ΔE3	2.975 (1.024)			
MHC-SWS ΔE_00_	5.705 (1.698)			
MHC-SWS ΔE1	1.616 (0.716)			
MHC-SWS ΔE2	2.597 (0.811)			
MHC-SWS ΔE3	3.35 (1.357)			
NHC-DPW ΔE_00_	4.226 (1.402)			
NHC-DPW ΔE1	1.984(1.682)			
NHC-DPW ΔE2	2.875 (1.118)			
NHC-DPW ΔE3	2.656 (0.839)			
NHC-COW ΔE_00_	4.405 (2.209)			
NHC-COW ΔE1	2.262 (0.817)			
NHC-COW ΔE2	2.955 (0.684)			
NHC-COW ΔE3	3.17 (0.752)			
NHC-SWS ΔE_00_	5.409 (2.256)			
NHC-SWS ΔE1	2.531 (1.045)			
NHC-SWS ΔE2	2.473 (0.981)			
NHC-SWS ΔE3	4.802 (2.044)			
SNFC-DPW ΔE_00_	4.246 (2.9)			
SNFC-DPW ΔE1	2.986 (1.821)			
SNFC-DPW ΔE2	2.197 (3.247)			
SNFC-DPW ΔE3	3.64 (2.458)			
SNFC-COW ΔE_00_	3.525(1.138)			
SNFC-COW ΔE1	2.755 (1.84)			
SNFC-COW ΔE2	3.3 (1.567)			
SNFC-COW ΔE3	3.605(1.681)			
SNFC-SWS ΔE_00_	4.596 (2.071)			
SNFC-SWS ΔE1	1.892(0.841)			
SNFC-SWS ΔE2	2.476 (0.929)			
SNFC-SWS ΔE3	3.595 (1.357)			

**Notes:**

The data are summarized as ‘median (interquartile range)’; IQR, interquartile range; *p*_*1*_ value: significance test result between measurements, *p*_2_ value; Intergroup PERMANOVA significance test result.

* Statistically significant (*p* < 0.05).

Color change values in the MHC-COW, microhybrid composite-colgate optic white; MHC-DPW, microhybrid composite-dentiste plus white; MHC-SWS, microhybrid composite-signal white system; NHC-COW, nanohybrid composite-colgate optic white; NHC-DPW, nanohybrid composite-dentiste plus white; NHC-SWS, nanohybrid composite-signal white system; SNFC-COW, supra-nano-filled composite-colgate optic white; SNFC-DPW, supra-nano-filled composite-dentiste plus white and SNFC-SWS, supra-nano-filled composite-signal white system composite-toothpaste subgroups at ΔE_00_: T_0_–T_1_; ΔE1: T_1_–T_2_; ΔE2: T_1_–T_3_; ΔE3: T_1_–T_4_ times were detected and statistically compared.

There were statistically significant differences among ΔE_00_, ΔE1, ΔE2 and ΔE3 values in terms of color change values (F = 70.19, *p*_*1*_ < 0.001). Color change measurements at ΔE_00_ were significantly higher than the measurements at ΔE1, ΔE2 ΔE3 periods (*p* < 0.001). In the nanohybrid composite-Signal White System and supra-nano-filled composite-Dentiste Plus White groups, color change measurements at ΔE1 were significantly higher than color change measurements at ΔE2, while color change measurements at ΔE1 were significantly lower in all other groups compared to color change measurements at ΔE2 (*p* < 0.001). Color change measurements at ΔE1 were significantly lower than color change measurements at ΔE3 (*p* < 0.001). While color change measurements at ΔE3 were significantly lower in the nanohybrid composite-Dentiste Plus White group compared to color change measurements at ΔE2, color change measurements at ΔE3 were significantly higher in all other groups compared to color change measurements at ΔE2 (*p* < 0.001). In addition, a statistically significant difference was found among the groups in terms of color change measurements (F = 2.46, *p*_*2*_ = 0.01). Color change measurements were significantly different in the nanohybrid composite-Signal White System group compared to the microhybrid composite-Dentiste Plus White and nanohybrid composite-Dentiste Plus White groups (*p* < 0.001). Except for the color change measurements at ΔE_00_, color change measurements were significantly higher in the supra-nano-filled composite-Dentiste Plus White group compared to the microhybrid composite-Dentiste Plus White group (*p* < 0.001). According to the results obtained from the study, the time*group interaction effect for color change measurements was not found statistically significant (F = 1.17; *p* = 0.25).

In Table 5, the results of the multiple linear regression model created to determine the independent predictors of color change results are given. Since the stepwise variable selection method was used while creating the model, all variables were excluded from the model at the time of ΔE_00_. It was observed that microhybrid composite was a reducing factor in the whitening effect of toothpastes at ΔE1 (T1–T2) time (β 95% CI −0.665 [−1.190 to −0.141]). Similarly, microhybrid composite was a reducing factor in the whitening effect of toothpastes at ΔE2 (T1–T3) time (β 95% CI −0.575 [−1.052 to −0.098]). At ΔE3 (T1–T4) time, there was only Dentiste Plus White toothpaste in the model as a result of variable selection, and it was observed that Dentiste Plus White was a factor that had a reducing effect on color change results (β 95% CI −0.547 [−1.074 to −0.019]).

**Table 5:** Multiple linear regression findings for color change results in each time period.

Time	Variable	*β*	SE	t- value	*p*-value	95% CI for *β*		R-squared
						Lower	Upper	
ΔE1 (T1–T2)	Constant	2.646	0.152	17.374	<0.001	2.343	2.948	0.067
	Composite = Microhybrid	−0.665	0.264	−2.523	0.013	−1.190	−0.141	
ΔE2 (T1–T3)	Constant	3.051	0.139	22.014	<0.001	2.776	3.326	0.061
	Composite = Microhybrid	−0.575	0.240	−2.395	0.019	−1.052	−0.098	
ΔE3 (T1–T4)	Constant	3.681	0.153	24.018	<0.001	3.376	3.985	0.046
	Toothpaste = Dentiste plus white	−0.547	0.265	−2.060	0.042	−1.074	−0.019	

**Note:**

SE, standard error; CI, confidence interval.

### Surface roughness change results

**Figure 2:**
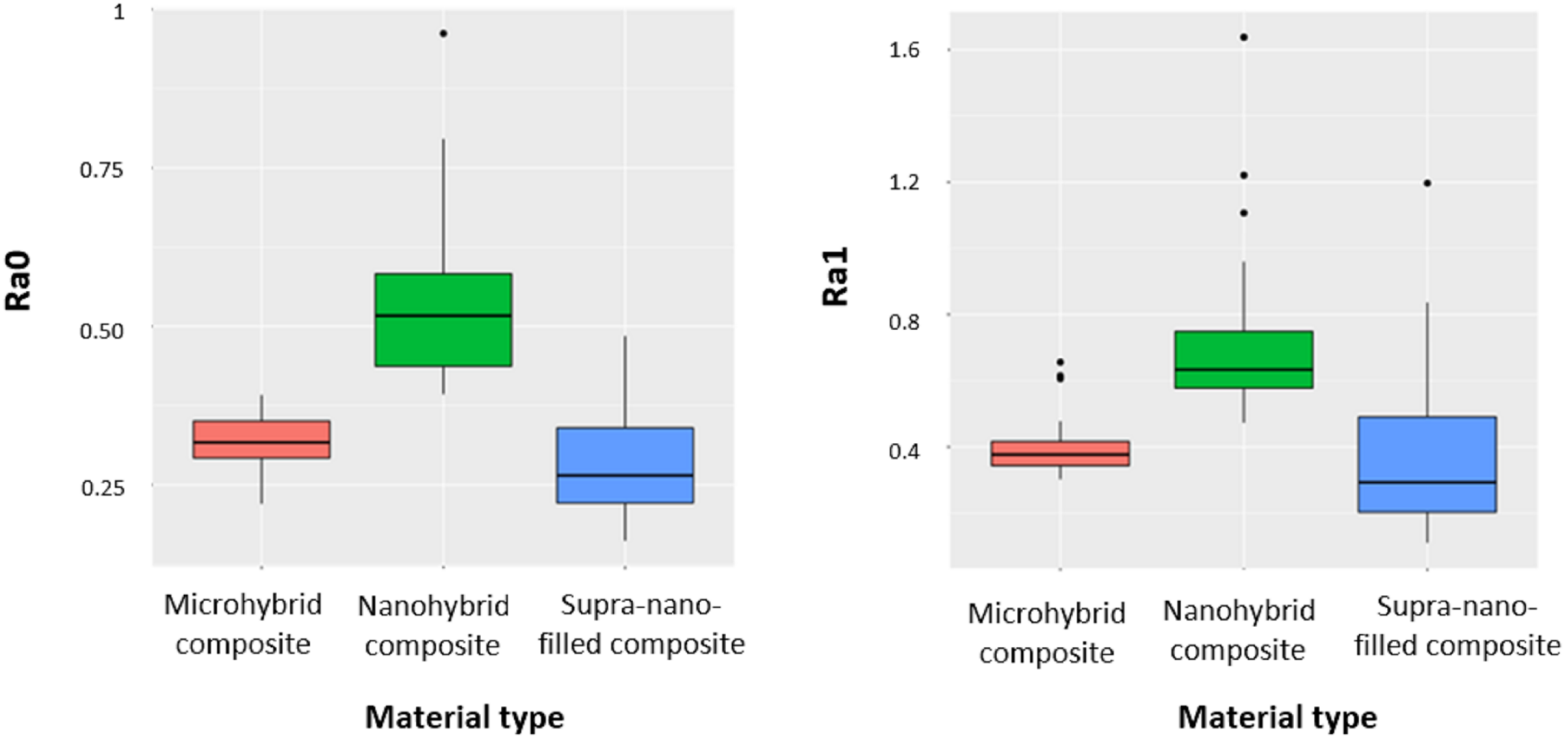
Changes for Ra_0_ and Ra_1_ values in the main material groups.

The surface roughness findings of the main groups before (Ra_0_) and after (Ra_1_) brushing are shown in Fig. 2. According to the research findings, there was a statistically significant difference between the main groups in terms of Ra_0_ and Ra_1_ values (*p* < 0.001). Ra_0_ and Ra_1_ values were significantly higher in the nanohybrid composite group compared to the microhybrid composite and supra-nano-filled composite groups (*p* < 0.001). In addition, while the Ra_0_ values were significantly higher in the microhybrid composite group compared to the supra-nano-filled composite group, no statistically significant difference was determined between the microhybrid composite and supra-nano-filled composite groups for the Ra_1_ value.

**Figure 3:**
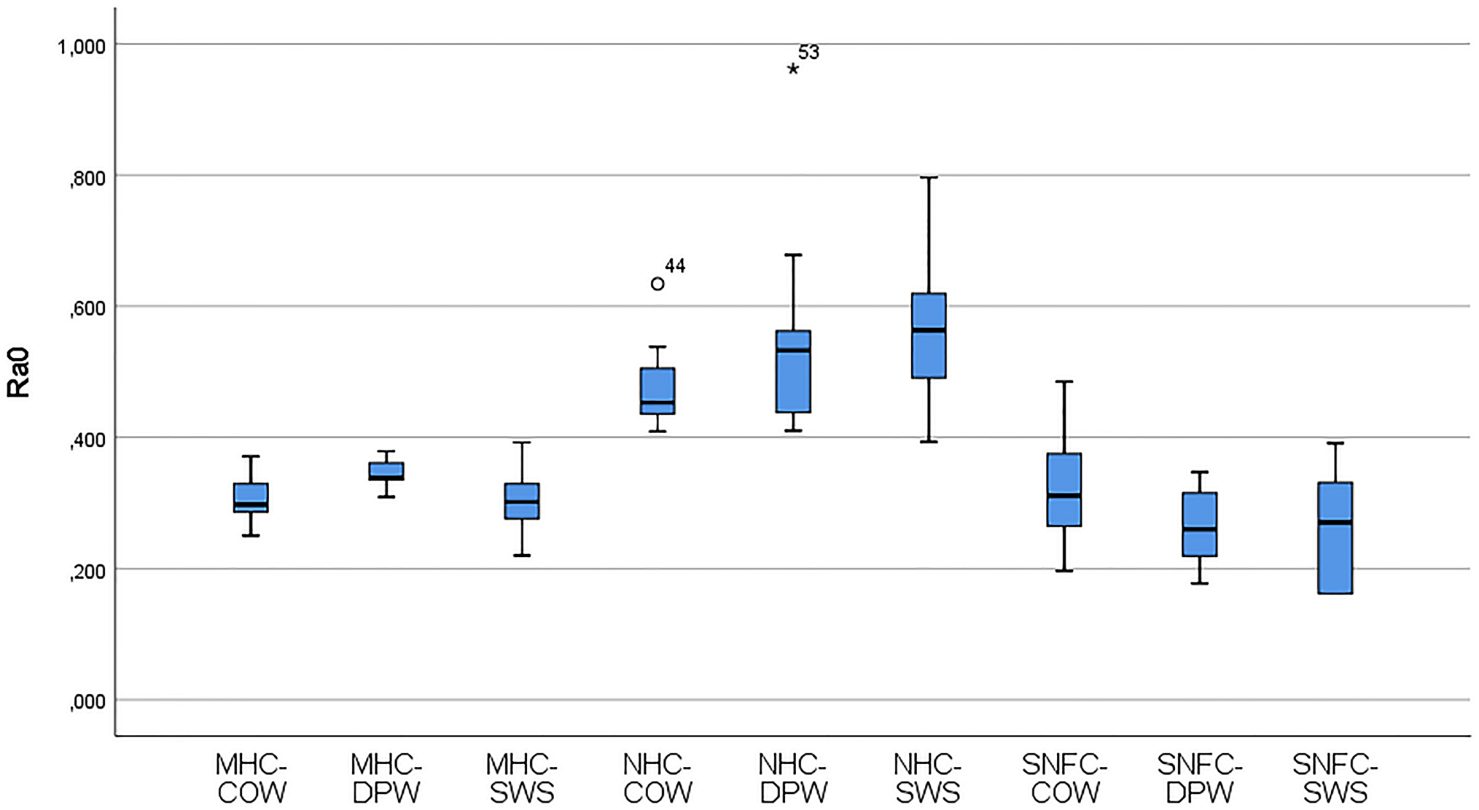
Box-plot graphs for Ra_0_ data of the MHC-COW, microhybrid composite-colgate optic white; MHC-DPW, microhybrid composite-dentiste plus white; MHC-SWS, microhybrid composite-signal white system; NHC-COW, nanohybrid composite-colgate optic white; NHC-DPW, nanohybrid composite-dentiste plus white; NHC-SWS, nanohybrid composite-signal white system; SNFC-COW, supra-nano-filled composite-colgate optic white; SNFC-DPW, supra-nano-filled composite-dentiste plus white; SNFC-SWS, supra-nano-filled composite-signal white system composite-toothpaste subgroups.

**Figure 4:**
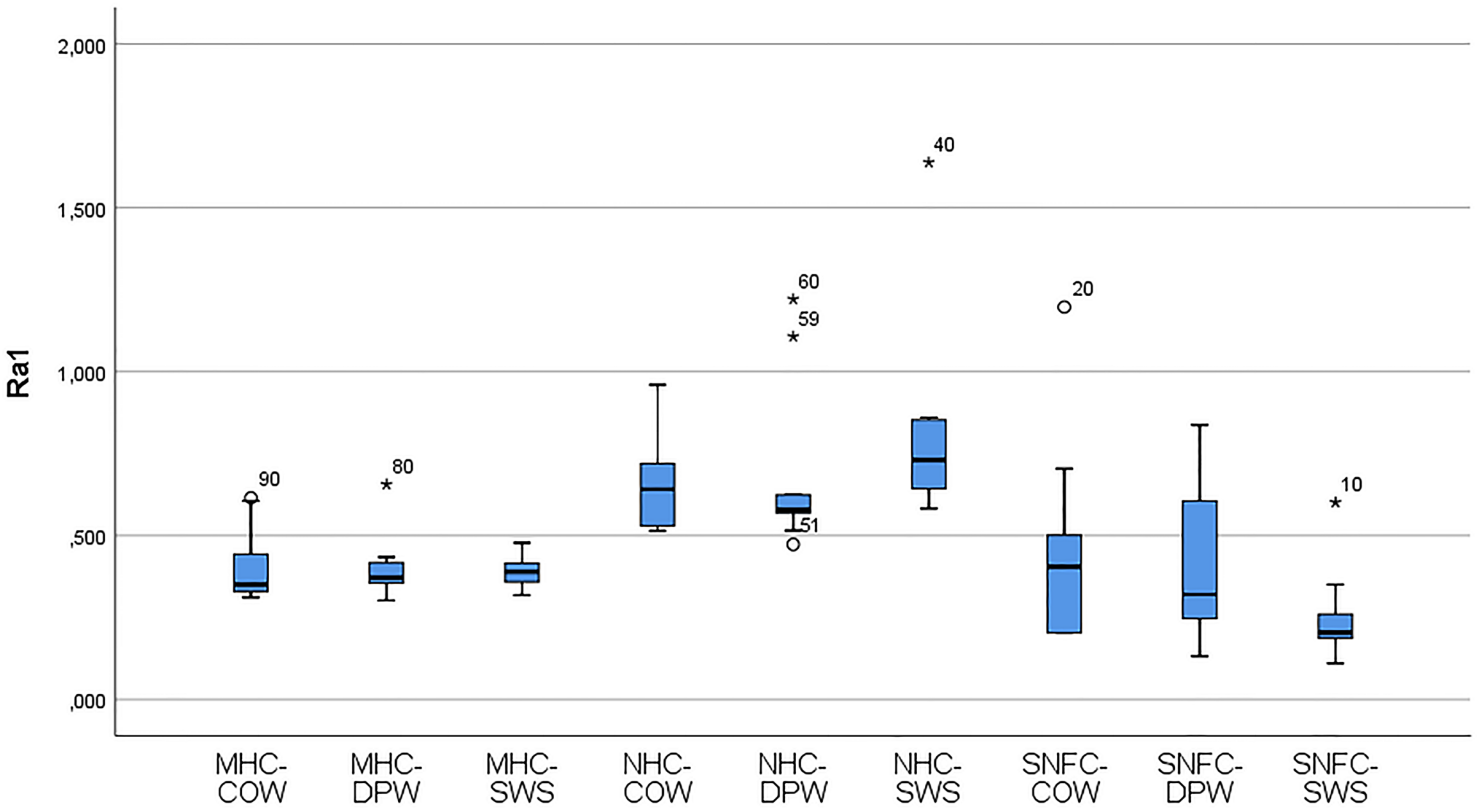
Box-plot graphs for Ra_1_ data of the MHC-COW, microhybrid composite-colgate optic white; MHC-DPW, microhybrid composite-dentiste plus white; MHC-SWS, microhybrid composite-signal white system; NHC-COW, nanohybrid composite-colgate optic white; NHC-DPW, nanohybrid composite-dentiste plus white; NHC-SWS, nanohybrid composite-signal white system; SNFC-COW, supra-nano-filled composite-colgate optic white; SNFC-DPW, supra-nano-filled composite-dentiste plus white; SNFC-SWS, supra-nano-filled composite-signal white system composite-toothpaste subgroups.

Table 6 shows the variation of the Ra_0_ and Ra_1_ values of the materials according to the toothpaste subgroups. The distribution of the variables of the Ra_0_ and Ra_1_ values of the composite-toothpaste subgroups is given with a box-plot graph (Figs. 3 and 4). According to the findings of the study, there was a statistically significant difference between the composite-toothpaste subgroups in terms of Ra_0_ and Ra_1_ values (*p* < 0.001). Ra_0_ and Ra_1_ values were significantly higher in the nanohybrid composite-Signal White System group compared to the microhybrid composite-Colgate Optic White, microhybrid composite-Dentiste Plus White, microhybrid composite-Signal White System, supra-nano-filled composite-Colgate Optic White, supra-nano-filled composite-Dentiste Plus White, and supra-nano-filled composite-Signal White System groups (*Statistical significance expressed in APA style with letters in *Table 6). However, there was no statistically significant difference between nanohybrid composite-Colgate Optic White, nanohybrid composite- Dentiste Plus White, and nanohybrid composite-Signal White System groups in terms of Ra_0_ and Ra_1_ values (*Statistical significance expressed in APA style with letters in *Table 6). In addition, the results of the repeated measurements for Ra_0_ and Ra_1_ values in the material-toothpaste subgroups indicated that Ra_1_ values were significantly higher in microhybrid composite-Colgate Optic White (*p* = 0.005), microhybrid composite-Dentiste Plus White (*p* = 0.04), microhybrid composite-Signal White System (*p* = 0.007), nanohybrid composite-Colgate Optic White (*p* = 0.009) and nanohybrid composite-Signal White System (*p* = 0.028) groups compared to Ra_0_ values.

**Table 6:** Changes in Ra_0_ and Ra_1_ values in composite-toothpaste subgroups.

Variable**	Groups*									*p*-value^#^	ES
	MHC-COW	MHC-DPW	MHC-SWS	NHC-COW	NHC-DPW	NHC-SWS	SNFC-COW	SNFC-DPW	SNFC-SWS		
Ra_0_	0.298^ab^ (0.036)	0.338^c^ (0.024)	0.302^ab^ (0.046)	0.453^d^ (0.062)	0.532^d^ (0.119)	0.564^d^ (0.115)	0.311^a^ (0.105)	0.26^b^ (0.089)	0.27^ab^ (0.152)	<0.001	2.82
Ra_1_	0.351^a^ (0.093)	0.372^a^ (0.061)	0.39^a^ (0.05)	0.641^b^ (0.165)	0.578^b^ (0.051)	0.73^b^ (0.198)	0.405^a^ (0.26)	0.32^a^ (0.31)	0.204^c^ (0.063)	<0.001	1.96
*p*-value^$^ (Ra_0_ *vs*. Ra_1_)	0.005	0.04	0.007	0.009	0.169	0.028	0.506	0.139	0.443		

**Notes:**

* There is a statistically significant difference in-group categories that do not contain the same letter in each row (Conover test; *p* < 0.05).

** Variables are summarized as ‘median (interquartile range)’;

# Kruskal Wallis H test.

$ Wilcoxon signed-rank test. ES, effect size.

Initial (Ra_0_) and final (Ra_1_) surface roughness values in the MHC-COW, microhybrid composite-colgate optic white; MHC-DPW, microhybrid composite-dentiste plus white; MHC-SWS, microhybrid composite-signal white system; NHC-COW, nanohybrid composite-colgate optic white; NHC-DPW, nanohybrid composite-dentiste plus white; NHC-SWS, nanohybrid composite-signal white system; SNFC-COW, supra-nano-filled composite-colgate optic white; SNFC-DPW, supra-nano-filled composite-dentiste plus white and SNFC-SWS, supra-nano-filled composite-signal white system composite-toothpaste subgroups were detected and statistically compared.

In Table 7, the results of the multiple linear regression model created to determine the independent predictors of Ra_0_ and Ra_1_ results are given. In the results of Ra_0_ (β 95% CI 0.231 [0.190 to 0.272]) and Ra_1_ (β 95% CI 0.333 [0.242 to 0.424]), nanohybrid composite was observed to be a factor with an enhancing effect on surface roughness.

**Table 7:** Multiple linear regression findings for Ra_0_ and Ra_1_ results.

Model	Variable	*β*	SE	t-value	*p*-value	95% CI for *β*		R-squared
						Lower	Upper	
Ra_0_	Constant	0.301	0.012	25.397	<0.001	0.278	0.325	0.590
	Composite = Nanohybrid	0.231	0.021	11.251	<0.001	0.190	0.272	
Ra_1_	Constant	0.385	0.027	14.539	<0.001	0.333	0.438	0.374
	Composite = Nanohybrid	0.333	0.046	7.253	<0.001	0.242	0.424	

**Note:**

SE, standard error; CI, confidence interval.

## Discussion

Whitening toothpastes, including those containing nanohydroxyapatite (n-HAP), are frequently preferred by patients due to their easy accessibility and low cost compared to clinical applications. The effects of these materials on the discoloration and surface roughness of restoration surfaces are not well known. In this study, the effects of toothpastes containing n-HAP (Dentiste Plus White, Dentiste, Thailand), perlite (Signal White System, Signal Unilever, London, UK), and hydrogen peroxide (Colgate Optic White) on the surface roughness and color stability of nanohybrid, microhybrid, and supra-nano-filled composites were evaluated and compared. When the ΔE1 and ΔE2 values of n-HAP-containing whitening toothpaste (Dentiste Plus White, Dentiste, Thailand) were compared in the supra-nano-filled composite group, it showed a similar effect only in the nanohybrid composite group that was applied perlite-containing whitening toothpaste (Signal White System, Signal Unilever, London, UK) (ΔE1 > ΔE2). In the nanohybrid composite-Dentiste Plus White group, ΔE3 values caused less color change compared to ΔE2 values, unlike the other groups. When the results were evaluated in terms of roughness, none of the whitening toothpastes caused a significant increase in the surface roughness of the supra-nano-filled composite group. Whitening toothpaste containing n-HAP did not cause a significant increase in the surface roughness of both the supra-nano-filled composite group and the nanohybrid composite group. Therefore, the null hypothesis of the present study was partially accepted.

The CIEDE2000 formula was used to evaluate the color changes in the study. This formula is a good indicator of human perceptible and acceptable color changes (Paravina, Pérez & Ghinea, 2019). Color changes were evaluated by making comparisons with 50:50% perceptibility and 50:50% acceptability thresholds. The perceptibility threshold for CIEDE2000 was taken as ΔE_00_ > 0.8 units and the clinical acceptability threshold as ΔE_00_ ≤ 1.8 units (Paravina et al., 2015). The color of esthetic restorative materials can change over time due to external factors. Roughness and discoloration on the surface of restorative materials and tooth lead to negative esthetic results (Rode et al., 2021; Yilmaz et al., 2021). Studies have emphasized that red wine, coffee, and tea are important external factors (Celik & Iscan Yapar, 2021; Omata et al., 2006; Temizci & Tunçdemir, 2021). In studies (Omata et al., 2006; Turker, Kocak & Aktepe, 2006; Farawati et al., 2019; Quek et al., 2018), coffee, tea, cola, and red wine were used as coloring solutions, while only coffee was used in others (Celik & Iscan Yapar, 2021; Temizci & Tunçdemir, 2021; Turgut et al., 2018). While Turker, Kocak & Aktepe (2006) found that red wine and tea were the beverages that produced the most coloration, Farawati et al. (2019) used wine, coffee, and tea as a coloring solution in their study, and coffee and wine produced significantly more coloration than tea, but did not show any significant difference from each other. According to some authors, coffee causes the most discolouration, followed by red wine and tea (Paolone et al., 2022). Manno et al. (2018) have identified coffee as the most popular beverage consumed by millions of people around the world every day. Therefore, the coffee solution was used as a colorant in the present study. The critical coloration period is the first 5 weeks, during which stain penetration can reach up to five microns (Ozkanoglu & Akin, 2020). In this study, the samples were immersed in coffee for 5 days (Celik & Iscan Yapar, 2021). In the ΔE_00_ data obtained after coloring with coffee, color changes occurred in all groups above the clinically acceptable threshold. When the data obtained after the application of whitening toothpastes were evaluated, no color change occurred above the clinically acceptable threshold in terms of ΔE1 values only in the microhybrid composite-Dentiste Plus White and microhybrid composite-Signal White System groups. The adsorption and absorption mechanisms occurring in the organic phase of the composite explain the coloration that occurs with coffee (Celik & Iscan Yapar, 2021). As a result of our study, the group that showed the most coloration after exposure to coffee was the microhybrid structured composite group, while the group that showed the least coloration was the supra-nano-structured composite group. In their study evaluating the color stability of microhybrid and nanohybrid composites, Ugurlu, Temel & Hepdeniz (2019) stated that the most colored samples in all subgroups belonged to the microhybrid composite group. In another study, Aydın et al. (2021) investigated the surface coloration changes of composite samples containing different types of fillers with polished surfaces. As a result, as in our study, the most colored group was the composite group containing microhybrid particles. The coloration differences between composites are closely related to the resin matrix and filler type (Erdemir, Yildiz & Eren, 2012). In their study examining the effects of various factors on the color of composites, Menon, Ganapathy & Mallikarjuna (2019) stated that the large filler particle size of the composite and the fact that the filler content is composed of different materials are important factors that increase the coloration. The fact that the microhybrid composite contains larger particles in the inorganic part compared to the other composites used in the study may be one of the factors explaining its higher coloration with coffee and lesser color change after the application of whitening toothpastes.

There are many whitening toothpastes on the market to remove or prevent discoloration on tooth surfaces. It is thought that substances such as calcium carbonate, perlite, calcium pyrophosphate, silicon dioxide, and hydrogen peroxide in the paste provide a whiter appearance by removing the chromophore and biofilm that cause coloration (Koc Vural et al., 2021). An innovative tooth-whitening component is hydroxyapatite (HAP) (Limeback, Meyer & Enax, 2023). *In vivo* and *in vitro* studies using HAP have generally investigated its remineralizing and desensitizing effects. Studies examining the teeth-whitening effect of HAP are limited. Shang, Kaisarly & Kunzelmann (2022) stated that, as a result of their *in vitro* study, the addition of n-HAP particles to toothpaste provided effective whitening. In their observational clinical study, Steinert et al. (2020) examined the effects of an oral gel containing microcrystalline hydroxyapatite on teeth whitening over the course of 4 weeks of twice-daily dental care. They claimed that the biomimetic alternative to existing whitening agents for routine dental care, microcrystalline hydroxyapatite, is a viable whitening ingredient for oral care formulations. Steinert et al. (2020) examined the impact of a toothpaste containing biomimetic zinc hydroxyapatite (HAP) on subjective metrics during a four-week at-home use in another observational trial. Patients in this study stated that using the HAP toothpaste resulted in smoother and whiter teeth. In a randomized, double-blinded controlled clinical trial (Woo et al., 2014), 85 individuals with tooth discolorations were randomly selected to use one of three toothpastes containing either hydroxyapatite, hydrogen peroxide, or no active ingredient (placebo toothpastes). The patients were examined at the start of treatment and 1, 2, and 3 months later. This study found that toothpaste containing hydrogen peroxide was significantly more effective at lightening teeth than hydroxyapatite and placebo toothpastes (Woo et al., 2014). In our study, Dentiste Plus White toothpaste containing silicon dioxide and n-HAP showed the highest difference in color change analyzed at the end of the first brushing simulation after coloring with coffee (ΔE1). This change was found to be significantly higher in the supra-nano-filled composite-Dentiste Plus White group than in the microhybrid composite-Dentiste Plus White group at all brushing time intervals. In the supra-nano-filled composite-Dentiste Plus White group, the color change value obtained after 7 days of brushing (ΔE1), which included the first brushing performed after coloring with coffee, was also higher than the value obtained after brushing completed after 15 days (ΔE2). These findings show that n-HAP in toothpaste can provide more effective whitening in the early period. In terms of ΔE2 values, the group that showed the most color change was Colgate Optic White toothpaste containing silicon dioxide and hydrogen peroxide. The group with the highest color change in terms of ΔE3 values was the nanohybrid composite group in which Signal White System containing perlite was applied. In addition, the nanohybrid composite-Signal White System group showed a significant difference in color change measurements compared to the microhybrid composite-Dentiste Plus White and nanohybrid composite-Dentiste Plus White groups. Perlite, which is widely used in whitening toothpastes, has been highlighted for its good stain removal and polishing properties (Joiner et al., 2002). According to the results of our study, the highest whitening effect was obtained with toothpastes containing silicon dioxide (Dentiste Plus White and Colgate Optic White) on days 7 and 15 of measurement, while the highest color change was observed only on day 30 with perlite-containing toothpaste. In another study, Joiner (2009) stated that silicon dioxide-based toothpastes have whitening properties, and this effectiveness can be increased with other whitening agents that can be added to the toothpaste. The prominent color change of Dentiste Plus White and Colgate Optic White containing silicon dioxide as a common material at different times may be due to the different activities of n-HAP and hydrogen peroxide, respectively, which are also part of their contents.

In our study, the restorative material with the least color change in terms of ΔE1 and ΔE2 values was a microhybrid composite, while the material with the most color change was a supra-nano-filled composite. It is thought that the different results of toothpastes on different restorative materials are related to the results of their interactions with the composites, as well as the abrasives contained in the toothpastes. Concerning this, Monaghan, Trowbridge & Lautenschlager (1992) reported that the formulations of composite resins were different, resulting in different degrees of whitening. They also stated that the rate of conversion of the resin matrix to polymer allowed some resins to be more exposed to the bleaching material, while others were less affected.

When the surface roughness values of the composites before and after brushing were examined, the surface roughness of all the samples increased in general. Toothpastes caused significant roughness in the microhybrid composite. The supra-nano-filled composite was not significantly affected by the toothpastes in terms of roughness. The reason toothpastes cause different surface roughness increases on composite surfaces is related to the structural differences of composites (Menon, Ganapathy & Mallikarjuna, 2019). Resin composites with different formulations have different surface roughness resistances (Hu, Marquis & Shortall, 2003). The friction and shear forces that occur on the surface of the restorative material as a result of brushing cause material loss. The roughest surface before and after brushing belonged to the nanohybrid composite group. The fact that the composite with the nanohybrid particle structure is rougher than the microhybrid composite with larger particles may depend on the type, shape, and degree of hardening of the inorganic fillers (Ruivo et al., 2019). Farghal & Elkafrawy (2020) obtained a similar result in their study: they found the surface roughness values of the microhybrid composite to be lower than those of the nanohybrid composite. They stated that due to the production technique, the microhybrid composite did not undergo inorganic particle separation and elution during polishing, and that large particles could be broken off from the nanohybrid composite.

This study has some limitations. One of them was that the coloring was created only with coffee. Although coffee is the most consumed beverage in the world, the effect of other external coloring factors should also be evaluated. Another limitation is that oral conditions (food or drinking habits, temperature changes, saliva, smoking, *etc*.) cannot be directly imitated, because the results of the study were obtained by *in vitro* experiments. To subject all materials to the same technique and avoid any potential bias due to differences in saliva structure, food or drinking habits, and so on, we chose to conduct *in vitro* research in standardized laboratory settings. This method provided comprehensive control over all variables. Furthermore, this technique has a clear ethical benefit because it does not put humans in danger. Studies are needed to evaluate the discoloration and surface roughness effects of whitening toothpastes containing n-Hap on colored restorations *in vivo*.

## Conclusions

Within the limitations of the present *in vitro* study, it can be concluded that while whitening toothpastes containing nanohydroxyapatite and hydrogen peroxide provided a high color change in the early period, toothpaste containing perlite provided a higher color change over a longer time. In addition, while none of the whitening toothpastes showed a significant increase in the roughness of the supra-nano-filled composite surface, the toothpaste containing nanohydroxyapatite did not cause a significant increase in the surface roughness of the nanohybrid composite either. The use of whitening toothpastes containing nanohydroxyapatite can provide a color change without creating significant roughness on the surface of nanohybrid and supra-nano-filled restorative materials in cases where extrinsic discoloration is expected to be removed within a short time.

## Supplemental Information

10.7717/peerj.15692/supp-1 Supplemental Information 1 Raw Data of Roughness Measurements. Ra0: Initial roughness measurements
Ra1: Roughness measurements of after brushing toothpastes
DPW: Nanohyroxiapatite containing toothpaste (Dentiste Plus White)
COW: Hydrogen peroxide containing toothpaste (Colgate Optic White)
SWS: Perlite containing toothpaste (Signal White System)

10.7717/peerj.15692/supp-2 Supplemental Information 2 Raw data of color change measurements. T0: Initial colour measurements
T1: After coffee coloring measurements
T2: Color measurement after brushing 7 days
T3: Color measurement after brushing 14 days
T4: Color measurement after brushing 30 days
DPW: Nanohyroxiapatite containing toothpaste (Dentiste Plus White)
COW: Hydrogen peroxide containing toothpaste (Colgate Optic White)
SWS: Perlite containing toothpaste (Signal White System)

## Additional Information and Declarations

### Data Availability

The following information was supplied regarding data availability: The raw data of measurements are available in the Supplemental Files.

## References

[ref-1] Addy M, Hughes J, Pickles MJ, Joiner A, Huntington E (2002). Development of a method in situ to study toothpaste abrasion of dentine. Comparison of 2 products. Journal of Clinical Periodontology.

[ref-2] Arslan AK, Yaşar Ş, Çolak C, Yoloğlu S (2018). WSSPAS: an interactive web application for sample size and power analysis with R using shiny. Turkiye Klinikleri Journal of Biostatistics.

[ref-3] Atai M, Nekoomanesh M, Hashemi SA, Amani S (2004). Physical and mechanical properties of an experimental dental composite based on a new monomer. Dental Materials.

[ref-4] Aydın N, Topçu FT, Karaoğlanoğlu S, Oktay EA, Erdemir U (2021). Effect of finishing and polishing systems on the surface roughness and color change of composite resins. Journal of Clinical and Experimental Dentistry.

[ref-5] Beun S, Glorieux T, Devaux J, Vreven J, Leloup G (2007). Characterization of nanofilled compared to universal and microfilled composites. Dental Materials.

[ref-6] Celik N, Iscan Yapar M (2021). Colour stability of stained composite resins after brushing with whitening toothpaste. International Journal of Dental Hygiene.

[ref-7] de Moraes Rego Roselino L, Tonani Torrieri R, Sbardelotto C, Alves Amorim A, Noronha Ferraz de Arruda C, Tirapelli C, de Carvalho Panzeri Pires-de-Souza F (2019). Color stability and surface roughness of composite resins submitted to brushing with bleaching toothpastes: an in situ study. Journal of Esthetic and Restorative Dentistry.

[ref-8] Dos Santos JH, Silva NL, Gomes MG, Paschoal MA, Gomes IA (2019). Whitening toothpastes effect on nanoparticle resin composite roughness after a brushing challenge: an in vitro study. Journal of Clinical and Experimental Dentistry.

[ref-9] Epple M, Meyer F, Enax J (2019). A critical review of modern concepts for teeth whitening. Dentistry Journal.

[ref-10] Erdemir U, Yildiz E, Eren MM (2012). Effects of sports drinks on color stability of nanofilled and microhybrid composites after long-term immersion. Journal of Dentistry.

[ref-11] Farawati FAL, Hsu SM, O’Neill E, Neal D, Clark A, Esquivel-Upshaw J (2019). Effect of carbamide peroxide bleaching on enamel characteristics and susceptibility to further discoloration. The Journal of Prosthetic Dentistry.

[ref-12] Farghal N, Elkafrawy H (2020). The effects of activated charcoal and calcium carbonate based toothpastes on surface properties of composite resin restorative materials. Egyptian Dental Journal.

[ref-13] Hu X, Marquis PM, Shortall AC (2003). Influence of filler loading on the two-body wear of a dental composite. Journal of Oral Rehabilitation.

[ref-14] Jin J, Xu X, Lai G, Kunzelmann KH (2013). Efficacy of tooth whitening with different calcium phosphate-based formulations. European Journal of Oral Sciences.

[ref-15] Joiner A (2009). A silica toothpaste containing blue covarine: a new technological breakthrough in whitening. International Dental Journal.

[ref-16] Joiner A, Pickles MJ, Matheson JR, Weader E, Noblet L, Huntington E (2002). Whitening toothpastes: effects on tooth stain and enamel. International Dental Journal.

[ref-17] Kim BI, Jeong SH, Jang SO, Kim KN, Kwon HK, Park YD (2006). Tooth whitening effect of toothpastes containing nano-hydroxyapatite. Key Engineering Materials.

[ref-18] Koc Vural U, Bagdatli Z, Yilmaz AE, Yalçın Çakır F, Altundaşar E, Gurgan S (2021). Effects of charcoal-based whitening toothpastes on human enamel in terms of color, surface roughness, and microhardness: an in vitro study. Clinical Oral Investigations.

[ref-19] Kocaagaoglu H, Aslan T, Gürbulak A, Albayrak H, Taşdemir Z, Gumus H (2017). Efficacy of polishing kits on the surface roughness and color stability of different composite resins. Nigerian Journal of Clinical Practice.

[ref-20] Lee YK (2005). Comparison of CIELAB ΔE(*) and CIEDE2000 color-differences after polymerization and thermocycling of resin composites. Dental Materials.

[ref-21] Limeback H, Meyer F, Enax J (2023). Tooth whitening with hydroxyapatite: a systematic review. Dentistry Journal.

[ref-22] Lu H, Stansbury JW, Nie J, Berchtold KA, Bowman CN (2005). Development of highly reactive mono-(meth)acrylates as reactive diluents for dimethacrylate-based dental resin systems. Biomaterials.

[ref-23] Luo MR, Cui G, Rigg B (2001). The development of the CIE, 2000 colour-difference formula: CIEDE2000. Color Research and Application.

[ref-24] Manno SHC, Manno FAM, Ahmed I, Ahmed R, Shu L, Li L, Xu S, Xie F, Li VW, Ho J, Cheng SH, Lau C (2018). Spectroscopic examination of enamel staining by coffee indicates dentin erosion by sequestration of elements. Talanta.

[ref-25] Menon A, Ganapathy DM, Mallikarjuna AV (2019). Factors that influence the colour stability of composite resins. Drug Invention Today.

[ref-26] Monaghan P, Trowbridge T, Lautenschlager E (1992). Composite resin color change after vital tooth bleaching. The Journal of Prosthetic Dentistry.

[ref-27] Moszner N, Klapdohr S (2004). Nanotechnology for dental composites. International Journal of Nanotechnology.

[ref-28] Omata Y, Uno S, Nakaoki Y, Tanaka T, San H, Yoshid S, Sidhu SK (2006). Staining of hybrid composites with coffee, oolong tea, or red wine. Dental Materials Journal.

[ref-29] Onuma K, Yamagishi K, Oyane A (2005). Nucleation and growth of hydroxyapatite nanocrystals for nondestructive repair of early caries lesions. Journal of Crystal Growth.

[ref-30] Ozkanoglu S, Akin EGG (2020). Evaluation of the effect of various beverages on the color stability and microhardness of restorative materials. Nigerian Journal of Clinical Practice.

[ref-31] Paolone G, Formiga S, De Palma F, Abbruzzese L, Chirico L, Scolavino S, Goracci C, Cantatore G, Vichi A (2022). Color stability of resin-based composites: staining procedures with liquids-A narrative review. Journal of Esthetic and Restorative Dentistry.

[ref-32] Paravina RD, Ghinea R, Herrera LJ, Bona AD, Igiel C, Linninger M, Sakai M, Takahashi H, Tashkandi E, Perez Mdel M (2015). Color difference thresholds in dentistry. Journal of Esthetic and Restorative Dentistry.

[ref-33] Paravina RD, Pérez MM, Ghinea R (2019). Acceptability and perceptibility thresholds in dentistry: a comprehensive review of clinical and research applications. Journal of Esthetic and Restorative Dentistry.

[ref-34] Perez MM, Hita-Iglesias C, Ghinea R, Yebra A, Pecho OE, Ionescu AM, Crespo A, Hita E (2016). Optical properties of supra-nano spherical filled resin composites compared to nanofilled, nano-hybrid and micro-hybrid composites. Dental Materials Journal.

[ref-35] Quek SHQ, Yap AUJ, Rosa V, Tan KBC, Teoh KH (2018). Effect of staining beverages on color and translucency of CAD/CAM composites. Journal of Esthetic and Restorative Dentistry.

[ref-36] Rode SM, Sato TDP, Matos FS, Correia AMO, Camargo SEA (2021). Toxicity and effect of whitening toothpastes on enamel surface. Brazilian Oral Research.

[ref-37] Roselino LDMR, Cruvinel DR, Chinelatti MA, Pires-de-Souza FDCP (2013). Effect of brushing and accelerated ageing on color stability and surface roughness of composites. Journal of Dentistry.

[ref-38] Ruivo MA, Pacheco RR, Sebold M, Giannini M (2019). Surface roughness and filler particles characterization of resin-based composites. Microscopy Research and Technique.

[ref-39] Sarembe S, Enax J, Morawietz M, Kiesow A, Meyer F (2020). In vitro whitening effect of a hydroxyapatite-based oral care gel. European Journal of Dentistry.

[ref-40] Schlafer S, Poulsen PN, Johansen J, Trap L, Leite FRM (2021). The whitening effect of single brushing with blue-covarine containing toothpaste-A randomized controlled trial. Journal of Dentistry.

[ref-41] Shang R, Kaisarly D, Kunzelmann KH (2022). Tooth whitening with an experimental toothpaste containing hydroxyapatite nanoparticles. BMC Oral Health.

[ref-42] Sivakumar JS, Prasad AS, Soundappan S, Ragavendran N, Ajay R, Santham K (2016). A comparative evaluation of microleakage of restorations using silorane-based dental composite and methacrylate-based dental composites in Class II cavities: an *in vitro* study. Journal of Pharmacy & Bioallied Sciences.

[ref-43] Steinert S, Kuchenbecker J, Meyer F, Simader B, Zwanzig K, Enax J (2020). Whitening effects of a novel oral care gel with biomimetic hydroxyapatite: a 4-week observational pilot study. Biomimetics.

[ref-44] Steinert S, Zwanzig K, Doenges H, Kuchenbecker J, Meyer F, Enax J (2020). Daily application of a toothpaste with biomimetic hydroxyapatite and its subjective impact on dentin hypersensitivity, tooth smoothness, tooth whitening, gum bleeding, and feeling of freshness. Biomimetics.

[ref-45] Temizci T, Tunçdemir MT (2021). Effects of bleaching agents on surface roughness and color change of discolored resin composites. Turkiye Klinikleri Journal of Dental Sciences.

[ref-46] Tinastepe N, Malkondu O, Iscan I, Kazazoglu E (2021). Effect of home and over the contour bleaching on stainability of CAD/CAM esthetic restorative materials. Journal of Esthetic and Restorative Dentistry.

[ref-47] Tokuyama Dental (2011). Tokuyama dental corporation technical report. https://www.tokuyama-us.com/wp-content/uploads/resources/Technical-Report_ESQ-3.pdf.

[ref-48] Turgut S, Kılınç H, Ulusoy KU, Bagis B (2018). The effect of desensitizing toothpastes and coffee staining on the optical properties of natural teeth and microhybrid resin composites: an in-vitro study. BioMed Research International.

[ref-49] Turker SB, Kocak A, Aktepe E (2006). Effect of five staining solutions on the colour stability of two acrylics and three composite resins based provisional restorations. The European Journal of Prosthodontics and Restorative Dentistry.

[ref-50] Ugurlu M, Temel UB, Hepdeniz OK (2019). Color stability of microhybrid and nanofilled composite resins: effect of surface sealant agents containing different filler content. The Journal of Contemporary Dental Practice.

[ref-51] Vaz VTP, Jubilato DP, Oliveira MRM, Bortolatto JF, Floros FC, Dantas AAR, Oliveira Junior OB (2019). Whitening toothpaste containing activated charcoal, blue covarine, hydrogen peroxide or microbeads: which one is the most effective?. Journal of Applied Oral Science.

[ref-52] Woo GJ, Kim EK, Jeong SH, Song KB, Goo HJ, Jeon ES, Choi YH (2014). Comparison of the whitening effect of toothpastes containing 0.25% hydroxyapatite and 0.75% hydrogen peroxide. Journal of Korean Academy of Oral Health.

[ref-53] Yagin B, Yagin FH, Gozukara H, Colak C (2021). A web-based software for reporting guidelines of scientific researches. The Journal of Cognitive Systems.

[ref-54] Yilmaz MN, Gul P, Unal M, Turgut G (2021). Effects of whitening toothpastes on the esthetic properties and surface roughness of a composite resin. Journal of Oral Science.

[ref-55] Yu H, Cheng SL, Jiang NW, Cheng H (2018). Effects of cyclic staining on the color, translucency, surface roughness, and substance loss of contemporary adhesive resin cements. Journal of Prosthetic Dentistry.

